# Surgery With Arterial Resection for Hilar Cholangiocarcinoma: Protocol for a Systematic Review and Meta-analysis

**DOI:** 10.2196/31212

**Published:** 2021-10-05

**Authors:** Artur Rebelo, Jörg Ukkat, Johannes Klose, Ulrich Ronellenfitsch, Jörg Kleeff

**Affiliations:** 1 Department of General, Visceral, Vascular and Endocrine Surgery University Hospital Halle (Saale) Martin-Luther-University Halle-Wittenberg Halle/Saale Germany

**Keywords:** meta-analysis, cholangiocarcinoma, arterial resection, surgery, vascular resections, cardiology, outcomes, mortality, morbidity, perioperative, cancer, tumor, liver, liver cancer

## Abstract

**Background:**

In light of recent advances in multimodality treatment, an analysis of vascular resection outcomes in surgery for hilar cholangiocarcinoma is lacking.

**Objective:**

The aim of this meta-analysis is to summarize the currently available evidence on outcomes of patients undergoing arterial resection for the treatment of hilar cholangiocarcinoma.

**Methods:**

A systematic literature search in the databases PubMed/MEDLINE, Cochrane Library, and CINAHL, and the trial registries ClinicalTrials.gov and the World Health Organization International Clinical Trials Registry Platform will be carried out. Predefined outcomes are mortality (100-day and in-hospital), morbidity (Clavien-Dindo classification, any type of complication), vascular complications (thrombosis or stenosis of the portal vein or hepatic artery, pseudoaneurysms), liver failure, postoperative bleeding, duration of surgery, reoperation rate, length of hospital stay, survival time, actuarial survival (2-, 3-, and 5-year survival), complete/incomplete resection rates, histologic arterial invasion, and lymph node positivity (number of positive lymph nodes and lymph node ratio).

**Results:**

Database searches will commence in December 2020. The meta-analysis will be completed by December 2021.

**Conclusions:**

Our findings will enable us to present the current evidence on the feasibility, safety, and oncological effectiveness of surgery for hilar cholangiocarcinoma with arterial resection. Our data will support health care professionals and patients in their clinical decision-making.

**Trial Registration:**

PROSPERO 223396; https://www.crd.york.ac.uk/prospero/display_record.php?RecordID=223396

**International Registered Report Identifier (IRRID):**

DERR1-10.2196/31212

## Introduction

Cholangiocarcinoma has an estimated incidence of 1 to 2 per 100,000 persons per year [1] and constitutes the second most common primary hepatic malignancy [2]. The effect of systemic treatment is limited in the majority of patients, and surgery with complete removal of the tumor is the only option offering a chance of cure or at least of long-term freedom from the tumor. In operated patients, a 20% to 30% 5-year overall survival can be achieved [3,4]. Most cholangiocarcinomas arise in the area of the bile duct bifurcation. They are commonly referred to as hilar cholangiocarcinoma or Klatskin tumor [5]. Due to the proximity of vascular structures to the bile duct bifurcation, tumor invasion of the portal vein, the proper hepatic artery, or the contralateral hepatic artery (ie, a tumor arising from the left bile duct invading the right hepatic artery) occurs in a relevant proportion of cases.

Vascular and especially arterial resection and reconstruction during surgical removal of hilar cholangiocarcinoma is a debated issue. Although it is the only way of facilitating complete resection in case of vascular invasion, there are concerns of high postoperative morbidity and mortality following vascular reconstruction, including hemorrhage and liver failure, which might offset the survival advantage gained from complete tumor removal. However, thanks to technical improvements in microvascular anastomoses and to a growing experience with liver transplants in many centers, the surgical approaches for hilar cholangiocarcinoma have generally become more aggressive in recent years, and thus, the number of studies assessing feasibility, safety, and oncological effectiveness of arterial resection and reconstruction has grown [6-10].

To summarize the currently available evidence on the topic, we plan to conduct a systematic review with meta-analysis.

## Methods

The literature search and data analysis will be conducted in accordance with the PRISMA (Preferred Reporting Items for Systematic Reviews and Meta-Analyses) guidelines [11]. The study has been registered in the PROSPERO database (ID 223396) [12].

### Search Strategy

The databases PubMed/MEDLINE, Cochrane Library, CINAHL, and the trial registries ClinicalTrials.gov and the World Health Organization International Clinical Trials Registry Platform will be searched through their respective online search engines. The search will be performed on studies published between database inception and a defined search date. The search strategies used in the individual databases are displayed in Multimedia Appendix 1. Furthermore, the reference lists of the included studies will be manually searched to find relevant articles. Abstracts and full-text reviews will be evaluated independently in a standardized manner by two authors to assess eligibility for inclusion or exclusion. Disagreements between reviewers will be resolved by consensus; if no agreement can be reached, a third reviewer will decide whether to include the study.

### Inclusion and Exclusion Criteria

Articles in the English, German, Spanish, Portuguese, and Italian language will be considered. Studies reporting resection of cholangiocarcinoma, both primary and secondary, in curative intention including resection of a segment of the hepatic artery with a control group of patients undergoing resection without arterial resection will be included. Review articles, case reports, case series with less than 5 patients, comments, and letters will not be included. The details of the study selection process will be summarized in a flowchart.

### Data Collection

Data from the individual included studies will be extracted separately by two authors and collected in a dedicated database. The following descriptive data will be documented for each selected study: first author, year of publication, inclusion period of the study, country where the study was conducted, sample size, and mean or median follow-up time. The distribution of the following patient and operation characteristics will be documented: age, gender, American Society of Anesthesiologists classification, Eastern Cooperative Oncology Group performance status, preoperative chemotherapy (yes/no, regimen), type of operation, type of vessel resection and reconstruction, duration of surgery, and blood loss. The following predefined outcomes will be extracted:

- Mortality (100-day and in-hospital)
- Morbidity (any complication according to the Clavien-Dindo classification [13] or another classification used in the respective study)
- Vascular complications (thrombosis, stenosis, or pseudoaneurysm of the portal vein or hepatic artery)
- Liver failure (as defined in the respective study)
- Postoperative bleeding (as defined in the respective study)
- Reoperation rate
- Mean and median survival
- 1-, 2-, 3-, and 5-year survival rates
- Proportion of macroscopically complete (R0), microscopically incomplete (R1), and macroscopically incomplete (R2) resection, and of patients without any resection upon surgery
- Histopathological tumor stage (pTNM)
- Proportion of patients with histologically confirmed arterial tumor invasion
- Mean and median of tumor-positive lymph nodes and of retrieved lymph nodes

For each study, the risk of bias will be assessed using the ROBINS-I (Risk of Bias in Nonrandomized Studies of Interventions) tool suggested by the Cochrane Collaboration [14]. An ideal randomized controlled trial on the pertinent research question will be conceived and emulated. The actual studies included in the meta-analysis will be compared with this emulated trial regarding their risk of bias in the following domains:

- Preintervention domains
  Bias due to confounding
  Bias in selection of participants into the study
- Intervention domain
  Bias in classification of interventions
- Postintervention domains
  Bias due to deviations from intended interventions
  Bias due to missing data
  Bias in measurement of the outcome
  Bias in selection of the reported result

For each domain, the tool foresees *signaling questions* whose response options are yes, probably yes, probably no, no, and no information. Based on the responses, the risk of bias for each domain will be judged as low, moderate, serious, critical, or no information. From the risk of bias for the single domains, an overall risk of bias for the study will be ascertained according to Table 1.

Because it is not expected that any randomized controlled trials will be identified for inclusion into the meta-analysis, no specific risk of bias assessment for such trials is planned.

**Table 1:** Bias judgement [14].

Overall risk of bias judgement	Interpretation	Criterion
Low risk of bias	The study is comparable to a well-performed randomized trial.	The study is judged to be at low risk of bias for all domains for this result.
Moderate risk of bias	The study appears to provide sound evidence for a nonrandomized study but cannot be considered comparable to a well-performed randomized trial.	The study is judged to be at low or moderate risk of bias for all domains.
Serious risk of bias	The study has one or more important problems.	The study is judged to be at serious risk of bias in at least one domain but not at critical risk of bias in any domain.

### Statistical Analysis

A meta-analysis with the following outcomes will be performed:

- Morbidity (any complication according to the Clavien-Dindo classification [13] or another classification used in the respective study)
- Vascular complications (thrombosis, stenosis, or pseudoaneurysm of the portal vein or hepatic artery)
- Liver failure (as defined in the respective study)
- Postoperative bleeding (as defined in the respective study)
- Reoperation rate
- 1-, 2-, 3-, and 5-year survival rates
- Proportion of macroscopically complete (R0), microscopically incomplete (R1), and macroscopically incomplete (R2) resection, and of patients without any resection upon surgery
- Histopathological tumor stage (pTNM)
- Proportion of patients with histologically confirmed arterial tumor invasion
- Mean and median of tumor-positive lymph nodes and of retrieved lymph nodes

The Review Manager (RevMan) software, version 5.3 (Cochrane Collaboration) will be used. The magnitude of the effect estimate will be visualized by forest plots. An odds ratio will be calculated for binary data and the weighted mean difference and relative difference of SD for continuous data. The 95% CI, heterogeneity, and statistical significance will be reported for each outcome. The chi-square and Kruskal-Wallis tests will be used for the evaluation of statistical significance. *P*<.05 will be considered statistically significant. When the studies do not report mean and SD, these will be calculated using the methods described by the guidelines of the Cochrane Collaboration [15] and Hozo et al [16]. As not all studies report hazard ratios, the survival analysis will be performed with weighted rates at the predefined time points previously listed. The outcome postoperative complications will be dichotomized (grade 1 and 2 vs grade 3a and higher according to the Clavien-Dindo classification). The incidence of severe complications (grade 3a and higher) per group will be determined and compared using the chi-square test and a forest plot. The histopathological tumor stage (pTNM) will be qualitatively described for the groups.

Subgroup analysis for patients with portal vein resection and patients who had undergone neoadjuvant chemotherapy prior to resection will be performed.

Sensitivity analyses will be conducted according to ascertained risk of bias as previously described. For these, all studies with a high/serious risk of bias will be excluded, and the analyses of the outcomes, as previously described, will be conducted.

## Results

Database searches will commence in December 2020. The meta-analysis will be completed by December 2021.

## Discussion

This systematic review with meta-analysis will synthetize all available evidence on feasibility, safety, and oncological effectiveness of arterial resection and reconstruction during surgical removal of hilar cholangiocarcinoma. It will be conducted according to the defined protocol presented here and will be reported following the recommendations stipulated in the PRISMA statement, thus ensuring the highest quality standards and minimizing the risk of possible bias [11]. The expected results will support health care professionals and patients with locally advanced hilar cholangiocarcinoma in their decision making. Specifically, we expect the results to show if concerns of high postoperative morbidity and mortality following vascular reconstruction, which might offset the survival advantage gained from complete removal of the tumor, are indeed justified.

## Appendix

Multimedia Appendix 1 Search strategy.

## Abbreviations

PRISMA: Preferred Reporting Items for Systematic Reviews and Meta-Analyses
ROBINS-I: Risk of Bias in Nonrandomized Studies of Interventions
