# Supplementary material for: Surgery With Arterial Resection for Hilar Cholangiocarcinoma: Protocol for a Systematic Review and Meta-analysis
Source: JMIR Res Protoc. 2021 Oct 5;10(10):e31212. doi: 10.2196/31212 (PMC8527376; doi:10.2196/31212)
Supplement: Multimedia Appendix 1 [file resprot_v10i10e31212_app1.docx]

Multimedia Appendix 1**. Search strategy.**

## PubMed

| **Hits** |
| --- |
| 4326 |

### P

| **"Cholangiocarcinoma"[Mesh] OR**  Cholangiocarcinoma*[tw] OR  ((Cholangiocellular[tw] OR  "bile duct*"[tw] OR  Klatskin*[tw] OR  biliar*[tw])  AND  **("Neoplasms"[Mesh] OR**  Neoplas*[tw] OR  Tumor*[tw] OR  Tumour*[tw] OR  Cancer*[tw] OR  Carcinoma*[tw] OR  malignancy[tw] OR  adenocarcinoma*[tw] OR  adenoma*[tw])) | 51666 |
| --- | --- |

### I

| ((**"Hepatic Artery"[Mesh] OR**  **"Portal Vein"[Mesh] OR**  "hepatic arter*"[tw] OR  "Portal vein*"[tw] OR  Vascular*[tw] OR  Venous*[tw] OR  Venae*[tw] OR  Vessel*[tw])  AND  (**"General Surgery"[Mesh] OR**  **"Surgical Procedures, Operative"[Mesh] OR**  **"Hepatectomy"[Mesh] OR**  surg*[tw] OR  Resect*[tw] OR  Remov*[tw] OR  excision*[tw] OR  operat*[tw] OR  Hepatectom*[tw])) | 496582 |
| --- | --- |

**String**

**P**

**1 (51666)**

("Cholangiocarcinoma"[MeSH Terms] OR "cholangiocarcinoma*"[Text Word] OR (("Cholangiocellular"[Text Word] OR "bile duct*"[Text Word] OR "klatskin*"[Text Word] OR "biliar*"[Text Word]) AND ("Neoplasms"[MeSH Terms] OR "neoplas*"[Text Word] OR "tumor*"[Text Word] OR "tumour*"[Text Word] OR "cancer*"[Text Word] OR "carcinoma*"[Text Word] OR "malignancy"[Text Word] OR "adenocarcinoma*"[Text Word] OR "adenoma*"[Text Word]))) AND (("Hepatic Artery"[MeSH Terms] OR "Portal Vein"[MeSH Terms] OR "hepatic arter*"[Text Word] OR "portal vein*"[Text Word] OR "vascular*"[Text Word] OR "venous*"[Text Word] OR "venae*"[Text Word] OR "vessel*"[Text Word])

**I**

**2 (496582)**

("General Surgery"[MeSH Terms] OR "surgical procedures, operative"[MeSH Terms] OR "Hepatectomy"[MeSH Terms] OR "surg*"[Text Word] OR "resect*"[Text Word] OR "remov*"[Text Word] OR "excision*"[Text Word] OR "operat*"[Text Word] OR "hepatectom*"[Text Word]))

**3**

**1 AND 2 (4326)**

## Cochrane Library

| **Hits** |
| --- |
| 25 |

### P

| [mh "Cholangiocarcinoma"] OR  "Cholangiocarcinoma*":ti,ab,kw OR  (("Cholangiocellular":ti,ab,kw OR  "bile duct*":ti,ab,kw OR  "Klatskin*":ti,ab,kw OR  "biliar*":ti,ab,kw)  AND  ([mh "Neoplasms"] OR  "Neoplas*":ti,ab,kw OR  "Tumor*":ti,ab,kw OR  "Tumour*":ti,ab,kw OR  "Cancer*":ti,ab,kw OR  "Carcinoma*":ti,ab,kw OR  "malignancy":ti,ab,kw OR  "adenocarcinoma*":ti,ab,kw OR  "adenoma*":ti,ab,kw)) | 1432 |
| --- | --- |

### I

| ([mh "Hepatic Artery"] OR  [mh "Portal Vein"] OR  "hepatic arter*":ti,ab,kw OR  "Portal vein*":ti,ab,kw OR  "Vascular*":ti,ab,kw OR  "Venous":ti,ab,kw OR  "Venae*":ti,ab,kw OR  "Vessel*":ti,ab,kw)  AND  ([mh "General Surgery"] OR  [mh "Surgical Procedures, Operative"] OR  [mh "Hepatectomy"] OR  "surg*":ti,ab,kw OR  "operat*":ti,ab,kw OR  "Resect*":ti,ab,kw OR  "Remov*":ti,ab,kw OR  excision*:ti,ab,kw OR  "Hepatectom*":ti,ab,kw) | 11986 |
| --- | --- |

## Web of Science Core Collection

| **Hits** |
| --- |
| 2786 |

### P

| "Cholangiocarcinoma*" OR  ("Cholangiocellular" OR  "bile duct*" OR  "Klatskin*" OR  "biliar*")  AND  ("Neoplas*" OR  "Tumor*" OR  "Tumour*" OR  "Cancer*" OR  "Carcinoma*" OR  "malignancy" OR  "adenocarcinoma*" OR  "adenoma*") | 37752 |
| --- | --- |

### I

| ("hepatic arter*" OR  "Portal vein*" OR  "Vascular*" OR  "Venous"  "Venae*" OR  "Vessel*")  AND  ("surg*" OR  "operat*" OR  "Resect*" OR  "Remov*" OR  "excision*" OR  "Hepatectom*") | 182592 |
| --- | --- |

## CINAHL

| **Hits** |
| --- |
| 422 |

### P

| "Cholangiocarcinoma*" OR  (("Cholangiocellular" OR  "bile duct*" OR  "Klatskin*" OR  "biliar*")  AND  ("Neoplas*" OR  "Tumor*" OR  "Tumour*" OR  "Cancer*" OR  "Carcinoma*" OR  "malignancy" OR  "adenocarcinoma*" OR  "adenoma*")) | 5874 |
| --- | --- |

### I

| ("hepatic arter*" OR  "Portal vein*" OR  "Vascular*" OR  "Venous"  "Venae*" OR  "Vessel*")  AND  ("surg*" OR  "operat*" OR  "Resect*" OR  "Remov*" OR  "excision*" OR  "Hepatectom*") | 43798 |
| --- | --- |

## Clinical Trial Gov

<http://www.clinicaltrials.gov/>

| **Hits** |
| --- |
| 69 |

### P

| Cholangiocarcinoma OR  ((Cholangiocellular OR  "bile duct" OR  Klatskin OR  biliar)  AND  (Neoplasm OR  Tumor OR  Tumour OR  Cancer OR  Carcinoma OR  malignancy OR  adenocarcinoma OR  adenoma)) |  |
| --- | --- |

### I

| ("hepatic artery" OR  "Portal vein" OR  Vascular OR  Venous OR  Venae OR  Vessel)  AND  (surgery OR  operation OR  Resection OR  Removal OR  excision OR  Hepatectomy) |  |
| --- | --- |

### Search Terms

|  |  | **Hits** |
| --- | --- | --- |
| **P** | (Cholangiocarcinoma OR ((Cholangiocellular OR "bile duct" OR Klatskin OR biliar) AND (Neoplasm OR Tumor OR Tumour OR Cancer OR Carcinoma OR malignancy OR adenocarcinoma OR adenoma)))  AND | 861 |
| **I** | ((EXPAND[Concept] "hepatic artery" OR EXPAND[Concept] "Portal vein" OR Vascular OR Venous OR Venae OR Vessel) AND (surgery OR operation OR Resection OR Removal OR excision OR Hepatectomy)) | 11771 |
